# How dentists in Egypt perceive their knowledge, attitudes, and barriers they face in providing oral healthcare to geriatric patients: a cross-sectional study

**DOI:** 10.1186/s12903-023-03690-9

**Published:** 2023-11-29

**Authors:** Mohamed Ashraf Hall, Rasha Ashmawy, Inas Karawia, Ramy Mohamed Ghazy

**Affiliations:** 1 Alexandria Dental Research Center, Ministry of Health and Population, Alexandria, Egypt; 2 Department of Clinical Research, Maamora Chest Hospital, MOHP, Alexandria, Egypt; 3 Pediatric and Community Dentistry Department, Faculty of Dentistry, Pharos University, Alexandria, Egypt; 4 Tropical Health Department, High Institute of Public Health, Alexandria University, Alexandria, Egypt

**Keywords:** Knowledge and attitudes, Service delivery, Health care services access, Egyptian dentists, Geriatric dentistry

## Abstract

**Background:**

Geriatric dentistry is an understudied area in dental schools in Egypt. Our study aimed to assess the knowledge and attitudes of Egyptian dentists regarding geriatric oral health and identify barriers to delivering dental care to geriatric patients.

**Methods:**

We conducted an anonymous online cross-sectional study in November and December 2022, targeting dentists with varying levels of experience working in different Egyptian institutions. A 30-item questionnaire assessed the respondent’s views on geriatric oral health, perceived knowledge, attitudes, and barriers. The Google form was distributed through emails and commonly used social media platforms.

**Results:**

A total of 421 dentists responded to this online questionnaire. Of the respondents, 44.9% were male, 45.0% were between 20 and 29 years old, and 31.5% worked in more than one dental setting. Multivariate analysis revealed that female sex negatively affected attitude β = -1.72 [95%CI,-2.43 – -1.11]. The proportion of older patients who visited the respondents’ clinics per day (11–30%) and more than 30% increased perceived knowledge [β = 1.01 (95%CI, 0.41 –1.62), β = 1.50 (95%CI, 0.71–2.22)] and attitude [β = 0.70 (95%CI, 0.06–1.40), β = 0.73 (95%, 0.13–1.61)] while decreased the perceived barriers [β = -1.10 (95%CI, -1.91 – -0.32)] respectively. On the other hand, years of experience increased perceived knowledge only after 5–10 years [β = 1.02 (95%CI, 0.04–2.10)] and after more than 10 years [β = 1.30 (95%CI, 0.21–2.70)]. Governmental work only increased perceived barriers [β = 1.33 (95%CI, 0.10–2.54)], while living in the middle and west delta decreased perceived barriers [β = -0.91 (95%CI, -2.12 – -0.01 and β = -1.33, (95%CI, -2.22 – -0.40) respectively].

**Conclusions:**

Our study highlights the need to improve the knowledge and attitudes of young dentists towards geriatric dentistry. Furthermore, working conditions in dental facilities, particularly in the government sector and Upper Egypt, need to be improved to reduce barriers to delivering dental care to geriatric patients.

**Supplementary Information:**

The online version contains supplementary material available at 10.1186/s12903-023-03690-9.

## Introduction

The medical field has made major advances, leading to higher quality care and development of the global population. As a result, the population of older adults is continuing to increase. According to the World Health Organization (WHO), older adults are those aged 65 years or above. Surprisingly, by 2050, more than half of the global population will be over 60 years old, as predicted [1]. The Department of Economic and Social Affairs of the United Nations (UN) reports that in 2022, there were 771 million people aged 65 years or older worldwide. This number is expected to increase to 994 million by 2030 and 1.6 billion by 2050 [2]. While this is a significant achievement for the medical field, it presents a challenge to many healthcare systems, especially in developing countries [3]. The majority of the older adult population has multiple systemic diseases, including diabetes mellitus, hypertension, dementia, kidney and liver disease, and cancers. Additionally, there is a general decline in the physical ability to perform daily activities [4]. The aging process affects the oral cavity, with the loss of teeth, decreased salivary flow, and increased periodontal diseases, as well as the presence of cancerous or precancerous lesions [5]. Poor oral health is also associated with general health in older people [6, 7]. Dental professionals must exercise heightened care and possess specialized knowledge because of the unique medical and oral conditions commonly found in older adults. This knowledge should be adequately taught at the undergraduate or postgraduate level [7].

The increased prevalence of root caries in the older population can be attributed to poor diet, drug-related xerostomia, and root exposure [8]. A systematic review published in 2014 found that the most common periodontal finding is plaque-related gingivitis with mild to moderate bone loss [9]. Dry mouth caused by salivary hypofunction and polypharmacy, including antidepressants and antihypertensive medications, is a frequently reported problem that has a negative impact on the oral and general quality of life of older adults patients [10]. Dental caries and periodontal diseases are more prevalent in older adults, and is often associated with untreated edentulism, which is linked to masticatory and nutritional deficiencies. Complete edentulism affects 21.9% of people over 74 years old in the United States of America (USA) and 39.6% in New Zealand [5], with physical, physiological, mechanical, and psychological factors playing a major role in the success of complete dentures [11].

In 1970, the term “geriatric dentistry” was first introduced in discussions about educating dental students to treat compromised older adults patients [12]. To evaluate the education provided in geriatric dentistry, a multinational online survey was conducted in 2020, which included 83 dental schools in 24 countries on 6 continents. The study found that geriatric dentistry was a mandatory part of the undergraduate curriculum in only 56 schools [13]. A systematic review in 2019 concluded that a lack of knowledge, experience, and time, as well as patient refusal of treatment, were significant barriers to delivering oral and dental care to the older population [14]. Several surveys have been conducted in various countries to assess the level of knowledge, perception, and attitudes towards geriatric dentistry among undergraduate students and dental practitioners. In a 2021 study conducted in Croatia regarding dentists’ opinions on oral health care for older patients, differences were observed between dentists who had undergone geriatric dentistry training during their education and those who had not [15]. In the Netherlands and Belgium, differences of opinion between dentists in the two countries were relatively limited [16]. In Isfahan, a study revealed that 86.5% of dentists had moderate knowledge, 2.6% had good knowledge, and almost 30% were unsatisfied with their knowledge and experience in treating older people [17]. Similar studies have also been conducted in the Netherlands [18], India [19, 20], Malaysia [21], Saudi Arabia [22], and Brazil [23].

In Egypt, the older adults’ population is estimated to be nearly 3.96 million as of March 2022 [24]. Nevertheless, there is a notable gap in the existing literature when it comes to evaluating the knowledge of Egyptian dentists in the field of geriatric dentistry. Therefore, this cross-sectional study aimed to assess the perceived knowledge, attitudes, and barriers to the delivery of oral care to geriatric patients among Egyptian dentists with different levels of experience and working in various institutions. The study would provide better information to effectively direct continuous dental education in this important field, achieve recommendations that may improve geriatric dental care, and identify barriers to its effective delivery.

## Methodology

### Study design

We conducted an anonymous online questionnaire-based cross-sectional study using a Google form between November and December 2022 to assess the perceived knowledge, attitude, and barriers to the delivery of oral care to geriatric patients among Egyptian dentists with different levels of experience working in various institutions. The form was sent to dentists through emails and commonly used social media platforms, including Facebook, Telegram, and WhatsApp. The study was approved by the Research Ethics Committee of the Egyptian Ministry of Health and Population on 16 November 2022 (Com No: 19-2022/18). The researcher followed the International Guidelines for Research Ethics and the World Medical Association Declaration of Helsinki (version 2013). Anonymity and confidentiality were maintained to ensure participant privacy. Prior to beginning the questionnaire, an informed consent form was provided to the dentists, explaining the study’s objective and stating that by completing the questionnaire, they were consenting to participate in the study. We followed the STROBE guidelines for reporting observational studies (Supplementary file 1).

### Sample size

Since no previously published study has addressed geriatric dentistry knowledge in Egypt, we assumed that 50% of dentists have sufficient knowledge about geriatric dentistry, and calculated the minimum required sample size to be 384 with a precision of 5% at a 95% confidence level using Epi-INFO version 7.2. Based on prior research, our sample was structured to be reflective of a total population of 76,843 dentists in Egypt [25].

#### Inclusion and exclusion criteria

Our target population consisted of actively practicing dentists working in various sectors, including governmental, private, and academic, within Egypt, who had internet access through smartphones or computer systems. We excluded undergraduate students and non-Egyptian participants from our study.

### Study population and sampling method

We used two sampling methods for this study. The first method was convenience sampling, where the link to the questionnaire was sent to dentists directly through emails and direct messages. The second method was snowball sampling, where the participating dentists were asked to share the link with their colleagues through social media groups.

### Study outcome

Our primary outcome was to assess the level of perceived knowledge, attitudes, and opinions on barriers to the delivery of care to older adult patients, relative to demographic data from the dentist (sex, age group, level of experience and education, and type of practice).

### Data collection tool

We used a self-administered, validated questionnaire in English language based on previous literature [15, 16]. The questionnaire was pilot-tested among a group of 20 participants who confirmed that they found the questions easy to understand. It consisted of 30 closed-ended questions arranged into three sections, with opinions rated on a five-category Likert-type scale (1 = totally disagree, 5 = totally agree).

The first section contained 9 closed-ended questions on participant demographics and professional data, including gender, year of graduation, educational level, workplace region, type of practice, years of experience, the proportion of older patients to the total number of patients, desire to attend a course or congress on the topic of geriatric dentistry, and specialty. The second section included 6 questions that addressed the oral health status of geriatric patients from the dentist’s perspective. The third section consisted of 15 items regarding respondents’ views on knowledge, attitudes, and barriers in providing oral care to older adults. There were 5 questions to assess dentist perceived knowledge, with a score between 5 and 25 points, 4 questions were used to assess attitudes, with a score between 4 and 20, and 6 questions were used to assess barriers, with a score between 6 and 30 (Supplementary file 2).

### Statistical analysis

The data obtained from the questionnaire were analyzed using the Statistical Package for the Social Sciences (SPSS version 25, IBM Corp, Armonk, New York, NY, USA). We presented categorical variables as frequencies and percentages and quantitative variables as mean and standard deviation or median and interquartile range. Multivariate regression models were used to estimate the associations between demographic factors and perceived knowledge, attitudes, and barriers outcomes. Assumptions were checked for linear relationships between the outcome variable and independent variables, normally distributed residuals, no multicollinearity among independent variables using Variance Inflation Factor (VIF) values, and homoscedasticity (similar variance of error terms across the values of the independent variables).

## Results

Table 1 illustrates that 421 dentists completed the online questionnaire, 44.9% of the participants were male, and 45.0% were aged between 20 and 29 years. Nearly 60% (59.9%) of the respondents were from Cairo & Alexandria, and 31.5% worked in more than one dental setting. Concerning work experience, 38.7% of the dentists had more than ten years of experience in dentistry, with 42% being general practitioners. When asked if they were interested in expanding their knowledge regarding geriatric dentistry, 68.2% of the participants agreed.

**Table 1:** Characteristics of respondent dentists (N = 421)

Variables	Frequency	percentages
Gender		
Males	189	44.9
Females	232	55.1
Age (years)		
20–29	193	45.8
30–39	115	27.3
40–49	78	18.5
50+	35	8.4
Workplace region		
Cairo & Alexandria	252	59.9
Middle delta	54	12.8
Upper Egypt	42	10.0
West Delta	73	17.3
Workplace category		
Private	45	10.7
Governmental	119	28.3
University	124	29.5
Mixed	133	31.5
Education		
Bachelor	233	55.3
Diploma/Master	105	24.9
PhD/Fellowship	83	19.8
Years of experience		
Less than 5 years	147	34.9
5–10 years	111	26.4
More than 10	163	38.7
Proportion of older patients /day		
<10%	148	35.2
11–30%	198	47
>30%	75	17.8
Specialty		
General dentist practitioner	177	42
Specialist	244	58
Desire to attend geriatric course		
Yes	287	68.2
No	134	31.8

**Fig. 1:**
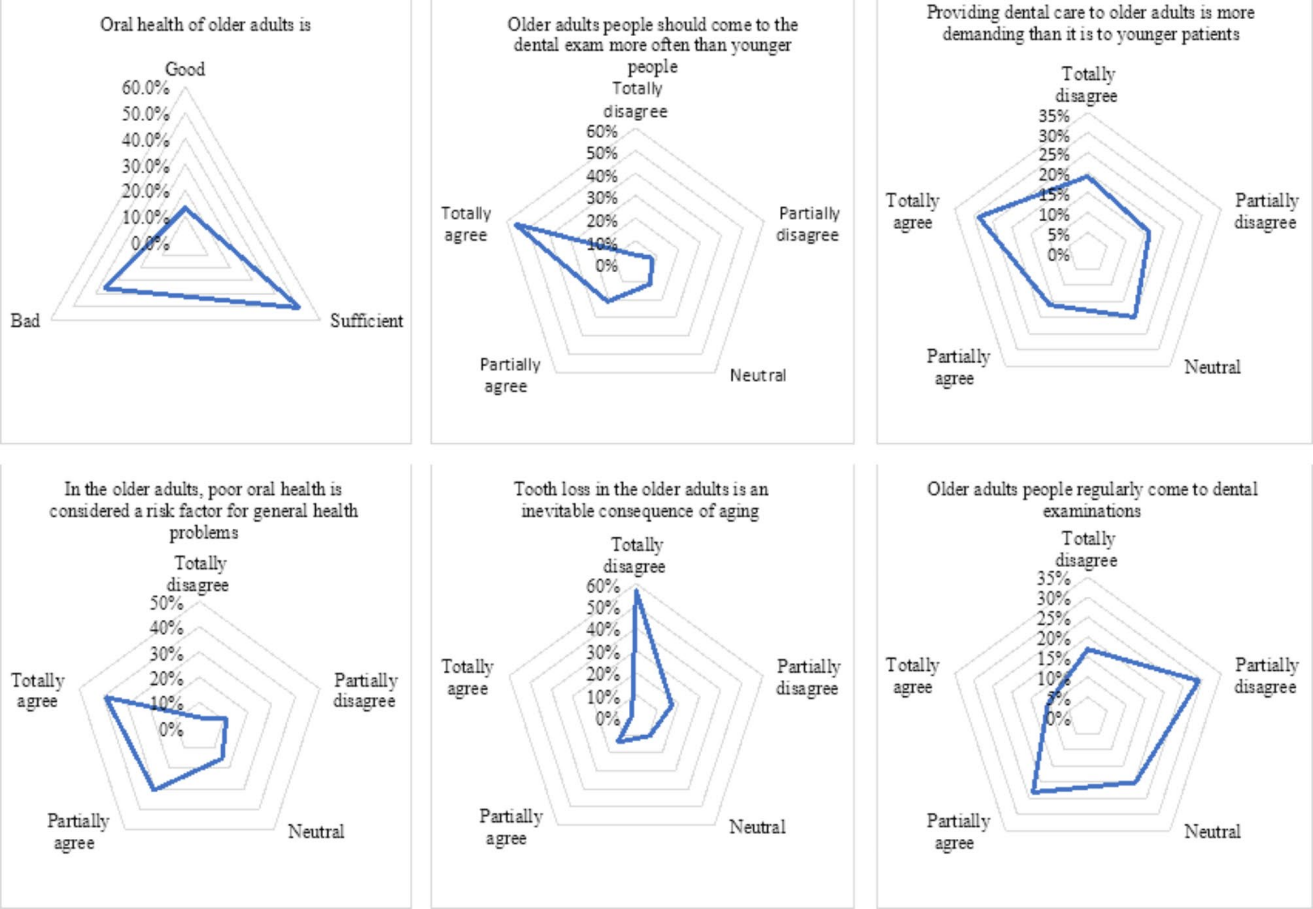
Respondents’ opinions regarding the oral health of the geriatric population

Figure 1 depicts the respondents’ opinions on geriatric oral health services, 51.0% of them believed it is sufficient, whereas 36% considered it to be poor. The following percentages of respondents either completely or partially agreed with the following statements: 33.0% believed that older adults regularly visit dental clinics, 77.0% agreed that the older population requires more examinations than younger individuals, 45.0% believed that providing dental care to older adults is more challenging than for younger people, 70.0% agreed that poor oral hygiene can lead to systemic complications, and 16.0% believed that tooth loss in older adults is an inevitable consequence of aging.

Table 2 presents the participants’ opinions on their perceived knowledge, attitudes, and barriers. A total of 52.3% either completely or partially agreed that they have sufficient knowledge about the adverse effects of medicines commonly used by geriatric patients. Moreover, 96% of the respondents believed that dental medicine studies should prioritize acquiring knowledge and skills in the treatment of older adults. Additionally, 47.3% agreed that they are ready to conduct regular home visits for dental check-ups for older adults. On the other hand, 72.9% agreed that providing oral healthcare to older people is challenging due to its complexity and practical obstacles. However, 61.8% stated that the dental offices where they practice are easily accessible to older adults with no significant barriers. Lastly, 17.4% of the respondents identified insufficient reimbursement for providing oral care to older adults as a barrier to their professional commitment to this specific patient group.

**Table 2:** Respondents’ answers to their perceived knowledge, attitudes, and barriers in providing oral care to older people

Questions	Totally Disagree	Partially Disagree	Neutral	Partially Agree	Totally Agree
	N (%)	N (%)	N (%)	N (%)	N (%)
K1—Physical, psychological, and social aspects may influence decision-making considering oral healthcare for older people	7(1.7)	9(2.1)	28(6.7)	91(21.6)	286(67.9)
K2—I have sufficient knowledge of the (adverse) effects of medicines commonly used by older people	31(7.4)	98(23.3)	72(17.1)	135(32.1)	85(20.2)
K3—I am capable of providing oral healthcare to cognitively impaired seniors	23(5.5)	78(18.5)	77(18.3)	158(37.5)	85(20.2)
K4—Dental medicine studies should pay more attention to the acquisition of sufficient knowledge and skills in the treatment of older people	2(0.5)	1(0.2)	14(3.3)	40(9.5)	364(86.5)
K5—Oral hygiene is a prerequisite for preventing oral health problems in older people	2(0.5)	2(0.5)	27(6.4)	55(13.1)	335(79.6)
A1—Each dentist is responsible for providing proper oral healthcare to older people who are unable to leave their home, but who have previously regularly come to their practice.	9(2.1)	110(26.1)	103(24.5)	153(36.3)	46(10.9)
A2—I am prepared to do a regular dental examination to an old and infirm person via a home visit	24.5(103)	16.9(71)	11.4(48)	123(29.2)	76(18.1)
A3—I have repeatedly experienced that at some point frail older people stopped coming for regular check-ups (appointments)	1.4(6)	02.9(12)	21.6(91)	115(27.3)	197(46.8)
A4—from the dentist’s point of view, treating older adults is not very challenging	13.5(57)	21.6(91)	97)23.0)	95(22.6)	81(19.2)
B1—Possibilities for referrals of older people with complex oral health problems to fellow specialists are limited	13.8(58)	34.2(144)	51(12.1)	97(23.0)	71(16.9)
B2—Providing oral healthcare to older people is difficult because of its complexity and practical obstacles	2.6(11)	011.6(49)	54(12.8)	155(36.8)	152(36.1)
B3—The reimbursement for providing oral health care to older people is insufficient	11.9(50)	35.6(150)	90(21.4)	102(24.2)	29(6.9)
B4—The institution (dental office) where I practice is easily accessible to older people (no major obstacles)	4.8(20)	24.2(102)	39(9.3)	111(26.4)	149(35.4)
B5—Usually, the provision of oral healthcare to older people involves various technical limitations	1.7(7)	6.4(27)	57(13.5)	195(46.3)	135(32.1)
B6—I find that insufficient reimbursement for the provision of oral healthcare to older people is a barrier to the professional commitment to this particular group of patients	44.9(189)	22.3(94)	65(15.4)	45(10.7)	28(6.7)

Table 3 demonstrates that the total perceived knowledge score was 20.9 ± 2.9 (maximum score of 25). Dentists who were 40 years old and above, held a Ph.D. or fellowship, worked in multiple settings (governmental, university, and private), had 10 years or more of experience, and had older adult patients representing more than 30% of their client base had significantly higher knowledge scores. The total attitude score was 13.5 ± 3.2 (maximum score of 20). Females had a significantly lower score than males (12.7 ± 3.1 vs. 14.6 ± 2.9, *p* < 0.001). Dentists who worked in more than one setting had a significantly higher attitude score than those who worked in other settings. The barriers score was 19.4 ± 3.6 (maximum score of 30). Dentists who worked in Upper Egypt reported facing significantly more barriers than others. Finally, dentists whose older adult clients represented less than 10% of their total client base reported significantly higher barrier scores than others.

**Table 3:** Perceived knowledge, attitude and barrier scores across different dentists’ characteristics

Studied variables	Perceived Knowledge Mean (SD)		Attitude Mean (SD)		Perceived Barrier Mean (SD)	
Total score	20.9 ( 2.9)		13.5 (3.2)		**19.4 (3.6)**	
Gender						
Male	21.1 (2.8)	t = 1.57 P = 0.21	14.6 (2.9)	t = 39.88 **P < 0.001**	19.5 (3.7)	t = 2.9 P = 0.58
Female	20.7 (2.9)		12.7 (3.1)		19.3 (3.6)	
Age (years)						
20–29	20.2 (2.9)^a^	F = 10.52 **P < 0.001**	13.8 (3.1)	F = 0.86 P = 0.46	19.1 (3.6)	F = 1.35 P = 0.26
30–39	20.9 (2.9)		13.2 (3.2)		19.7 (3.4)	
40–49	21.7 (2.6)^b^		13.4 (3.2)		19.5 (3.7)	
50+	22.5 (2.2)^b^		13.4 (3.3)		20.3 (3.9)	
Workplace region						
Cairo & Alexandria	20.8 (2.8)	F = 0.13 P = 0.9	13.4 (2.9)	F = 0.81 P = 0.492	19.7 (3.7)^a^	F = 3.83 **P = 0.01**
Middle Delta	20.9 (2.7)		13.9 (3.2)		18.8 (3.5)^b^	
Upper Egypt	21.1 (2.9)		13.5 (3.6)		20.1 (2.6)	
West Delta	20.8 (3.2)		13.9 (3.6)		18.4 (3.5)^b^	
Workplace category						
Private	21.1 (2.4)	F = 6.56 **P < 0.001**	13.9 (3.2)	F = 3.49 **P = 0.016**	18.9 (3.6)	F = 2.9 P = 0.07
Governmental	20.7 (3.1)		12.9 (3.4)^a^		20.1 (3.9)	
University	20.1 (2.6)^a^		13.4 (2.8)		18.9 (3.5)	
Mixed	21.6 (2.8)^b^		14.1 (3.1)^b^		19.4 (3.3)	
Education						
Bachelor	20.3 (2.7)^a^	F = 12.05 **P < 0.001**	13.8 (3.1)	F = 2.14 P = 0.119	19.2(3.7)	F = 0.90 P = 0.41
Diploma/Master	21.4 (3.0)^b^		13.0 (3.3)		19.8 (3.2)	
Ph.D./Fellowship	21.8 (2.7)^b^		13.7 (3.1)		19.4 (3.8)	
Years of experience						
Less than 5 years	19.8 (2.9)^a^	F = 18.52 **P < 0.001**	13.7 (3.1)	F = 1.62 P = 0.19	19.0 (3.8)	F = 2.08 P = 0.126
5–10 years	20.9 (2.6)^b^		13.9 (3.1)		19.3 (3.1)	
More than 10 years	21.8 (2.7)^b^		13.2 (3.2)		19.8 (3.7)	
Proportion of older patients /day						
<10%	20.2 (2.6)^a^	F = 8.38 **P < 0.001**	12.9 (3.0)	F = 4.37 P = 0.13	19.9 (3.7)^a^	F = 4.75 **P = 0.009**
11–30%	21.1 (3.1)^b^		13.8 (3.1)		18.9 (3.5)^b^	
>30%	21.7 (2.9)^b^		14.1 (3.4)		19.8 (3.4)	

Superscripts with different letters are statistically significant by pair-wise comparison, significant p-values are indicated in bold

Table 4 presents the detailed results of the multivariate linear regression analysis. In order to identify the factors affecting the dentists’ perceived knowledge, attitude, and barriers. Multivariate analysis revealed that female sex negatively affected attitude β = -1.72 [95%CI,-2.43 – -1.11]. The proportion of older patients who visited the respondents’ clinics per day (11–30%) and more than 30% increased perceived knowledge [β = 1.01 (95%CI, 0.41 –1.62), β = 1.50 (95%CI, 0.71–2.22)] and attitude [β = 0.70 (95%CI, 0.06–1.40), β = 0.73 (95%, 0.13–1.61)] while decreased the perceived barriers [ß =  -1.10 (95% CI, -1.91 – -0.32)]] respectively. On the other hand, years of experience increased perceived knowledge only after 5–10 years [ß = 1.02, (95% CI, 0.04–2.10)] and after more than 10 years [ß = 1.30, (95% CI, 0.21–2.70)]. Governmental work only increased perceived barriers [ß = 1.33, (95% CI, 0.10–2.54)], while living in the middle and west delta decreased perceived barriers [ß = -0.91, (95% CI, -2.12 – -0.01 and; ß = -1.33, (95% CI, -2.22 – -0.40). respectively].

**Table 4:** Multi linear regression of the respondents perceived knowledge, attitude, and barriers

Total (N = 421)	Knowledge ß [95% CI]	Attitude ß [95% CI]	Barriers ß [95% CI]
Intercept	19.61*[ 18.52; 20.60]	14.01*[12.81; 15.20]	19.82*[18.54; 21.23]
Gender			
Male	Ref.	Ref.	Ref.
Female	-0.04[-0.61; 0.54]	-1.72*[-2.43; -1.11]	-0.15[ -1.32; 0.21]
Age Category			
20–29	Ref.	Ref	Ref.
30–39	-0.52[-1.51; 0.53]	-0.64[-1.81; 0.53]	0.01[-1.32; 1.43]
40–49	-0.22[-1.24; 1.64]	0.14[ -1.52; 1.74]	-0.74[-2.53; 1.24]
50 or above	-0.92[− 0.61; 2.52]	-0.14[ -1.94; 1.63]	-0.05[ -2.21; 1.93]
Education			
Bachelor	Ref.	Ref	Ref.
Diploma/Master	0.33[-0.52; 1.12]	-0.60[ -1.53; 0.32]	-0.08[-1.13; 0.94]
Ph.D./Fellowship	0.62[ -0.34; 1.50]	0.34[ -0.73; 1.33]	-0.32[ -1.54; 0.82]
Workplace region			
Metropolis (Cairo/ Alex.)	Ref.	Ref.	Ref.
Middle Delta	-0.24[-1.01; 0.60]	0.30[-0.60; 1.24]	-0.91*[-2.12; -0.01]
Upper Egypt	-0.34[-1.23; 0.62]	-0.10[-1.24; 0.93]	0.41[-0.83; 1.62]
West Delta	-0.22[-0.92; 0.53]	0.34[-0.54; 1.13]]	-1.33*[ -2.22; -0.40]
Workplace category			
Private only	Ref.	Ref.	Ref.
Governmental only	-0.63[ -1.62; 0.42]	-0.12[-1.12; 0.91]	1.33*[0.10; 2.54]
University Only	-0.50[ -1.50; 0.52]	-0.132[-1.20; 0.92]	0.22[-1.21; 1.43]
Mixed	0.24[ -0.71; 1.23]	0.34[ -0.74; 1.42]	0.41[-0.83;2.92]
Years of experience			
Less than 5 years	Ref	Ref.	Ref.
5–10 years	1.02*[0.04; 2.10]	0.90[-0.22; 1.92]	0.24[ -1.12; 1.54]
More than 10 years	1.30*[ 0.21; 2.70]	-0.131[-1.72; 1.50]	1.13[ -0.84; 2.92]
Proportion of older patients /day			
Less than 10%	Ref.	Ref.	Ref.
11–30%	1.01*[0.41; 1.62]	0.70*[0.06; 1.40]	-1.10*[-1.91; -0.32]
More than 30%	1.50*[0.71; 2.22]	0.73*[ 0.13; 1.6]	-0.32[ -1.41; 0.74]
Model parameters			
F-statistics	4.55	3.8	12.11
DF	16, 404	16, 404	16, 404
p-value	< 0.00001	< 0.00001	0.0007

*Statistically significant. R: Reference category

## Discussion

Due to the epidemiological transition and the changes in mortality patterns, as well as the increasing life expectancy of the population, it has become increasingly necessary to pay attention to the health of older adults. The objective of the current study was to assess the perceived knowledge and attitudes of dentists and to identify the barriers they encounter in providing oral care to geriatric patients.

Over half of the respondents felt confident in their knowledge, while the majority believed that geriatric dentistry should receive more attention at the undergraduate and postgraduate levels. Dentist age, level of experience, working in multiple settings, and the proportion of older patients treated daily were associated with a higher level of perceived knowledge. Additionally, 78% of respondents believed that delivering dental care to older adults involved technical limitations. A more positive attitude among participating dentists was also associated with an increased proportion of older adults’ patients treated daily, while the highest level of perceived barriers was associated with working at governmental sector and in Upper Egypt. Nearly, 68% of study respondents agreed that they would like to attend a geriatric dentistry course or congress, which may be due to the fact that geriatric dentistry is not studied as a separate course during undergraduate education in Egypt. Moreover, 56% of participants completely agreed and 21% partially agreed that older adults should attend dental examinations more frequently than younger patients. This high percentage may be due to dentists’ understanding that with increasing age, the demand for oral health increases, but it is often neglected, especially in low-middle income countries like Egypt, where older patients may only visit the dentist if they have a problem or may never visit at all [26].

In the present survey, over half of the participants (52.3%) reported having adequate knowledge of the adverse effects associated with common geriatric medications. Additionally, an overwhelming majority of the respondents (96.0%) believed that dental schools should prioritize the acquisition of knowledge and skills related to the treatment of older adults. This is a higher percentage compared to the findings from previous studies conducted in Croatia (70.6%) and the Netherlands (84.4%) [15, 18]. The high percentage in the current study should be due to, as explained before, geriatric dentistry is not studied as a separate course in Egyptian dental schools. This is in accordance with the recommendations of Ettinger [27] who stated that dentists should provide treatment for older adults only after receiving adequate education and training in various aspects of aging and patient care. These findings highlight the need for dental schools to incorporate more geriatric dentistry training into their curricula to better equip future dentists with the necessary knowledge and skills to provide optimal care for older patients. In addition to the importance of continuing education and training for dentists to ensure they are equipped with the necessary skills and knowledge to provide appropriate care for older patients.

The findings of this study indicate that the level of experience is positively associated with a higher level of perceived knowledge among dentists in the treatment of older patients. This aligns with the results of a study of Bots-VanSpijker et al. [18] which also reported a positive correlation between dental experience and knowledge of geriatric dentistry. These findings suggest that dental professionals with more experience may have a better understanding of the unique oral health needs of older patients and may be better equipped to provide appropriate care. In contrast, Moreira et al. [23] reported that younger dentists expressed a higher level of knowledge of geriatric dentistry than their more experienced colleagues. It is possible that these conflicting findings may be due to differences in the study populations, methodology, or other factors. Further research may be needed to better understand the relationship between age, experience, and knowledge of geriatric dentistry among dental professionals.

The current study found a significant positive association between the proportion of geriatric patients seen by dentists in their daily practice and their perceived knowledge and attitudes towards geriatric dentistry, while also reporting lower barriers to providing care for older patients. These findings are consistent with previous studies, [15, 16, 18] which have also reported a positive correlation between the amount of experience treating older patients and knowledge of geriatric dentistry. Exposure to a larger number of geriatric patients can provide dentists with valuable experience and knowledge in this area, which can improve their ability to provide appropriate care. It is worth noting that Ettinger [27] recommended that dental students should gain adequate clinical experience working with older adults and medically compromised patients to develop the necessary skills in treatment planning and feel confident in providing care for these populations. These recommendations emphasize the importance of clinical exposure and experience in the education and training of dental professionals in geriatric dentistry.

According to a study of Malaysian dentists, the vast majority of governmental dentists were willing to provide in-home care for older adults regardless of their demographic characteristics, with dentists under the age of 30 being significantly more willing to provide the service [21]. In the current study, almost 47.0% of the participants believed that they were prepared to make regular home visits to older adults for dental examination, and 41.7% agreed that treating older adults is not very challenging. These findings are consistent with the findings of Bots-VantSpijker et al. [18] in the Netherlands (42%), while only 18.7% believed so in Croatia, which may be attributed to the difference in training received or wide availability of mobile dental unites in different countries. Moreira et al. [23] found that Brazilian female dentists showed a more positive attitude towards geriatric care, but the current study found that male dentists showed a more positive attitude, which may be explained by the higher number of patients they treated daily. However, no statistically significant difference was found between male and female dentists in the study done by Tahani et al. [17] in Iran.

In terms of the common barriers encountered in daily practice when treating older adults, the results demonstrated that an increased proportion of older patients visiting the respondent’s clinics per day (ranging from 11 to 30%) was associated with a decrease in perceived barriers. This finding is in line with the results of a study conducted by Bots-VanSpijker [18]. In the current study, 72.9% of the participants acknowledged that providing oral healthcare to older individuals is challenging due to its complexity and practical obstacles. This challenge can be attributed to the complex medical conditions prevalent in the older population, characterized by a higher incidence of disorders such as cardiovascular disease, dementia, and neurological diseases [18]. Furthermore, 61.8% stated that their workplace is easily accessible to older adults with no major obstacles. The fact that 40% of participants indicated there are limited options for referring older patients with complex dental issues to fellow specialists can be attributed to the absence of geriatric dentistry as a distinct specialty in Egypt. This situation often necessitates referring the patient to multiple specialists, which can be challenging, particularly for older patients. The findings of the current study concluded, in contrast to the findings obtained by the studies conducted in Croatia, Belgium, and the Netherlands [15, 16], that only 17.4% of the respondents found that insufficient reimbursement is a barrier to the professional commitment to this particular group of patients. This may be explained by differences in social or cultural beliefs between the dentists in different countries.

Dentists working in the governmental sector expressed a higher level of barriers with no statistically significant difference in their knowledge or attitude compared to their colleagues in other health sectors. This may be explained by the increased funding and more equipped facilities in the private and academic sectors compared to the governmental sector.

### Strengths and limitations

This study has some limitations. It was based on online questionnaire participation, which may have been influenced by participants’ interest in the subject or time spent on social media. Therefore, future paper-based studies are needed to confirm the findings of this study. However, a large percentage of the general population uses the internet and social media in Egypt (72.2%) [28], so we think that the population of this study is expected to be representative of dentists in Egypt. Another limitation is that undergraduate students were excluded from this study, so future studies are needed to assess their level of knowledge as they are the future of the dental profession. Lastly, the use of age categories for data collection, although practical, may not facilitate an in-depth analysis of age-related trends or relationships. On the other hand, this study is the first to investigate geriatric dental care in Egypt, and it utilized a valid questionnaire that enhance the internal consistency of the study findings.

## Conclusions

In our study, we observed that a higher level of knowledge was linked to greater experience and the proportion of older adult patients treated daily. Moreover, this enhanced knowledge was also associated with a more positive attitude. However, female dentists showed a less positive attitude regarding dental care for older adults. Nearly all respondents believed that dental schools should emphasize the acquisition of knowledge and skills in the treatment of older adults. Approximately half of the respondents expressed willingness to conduct regular home visits for dental examinations of older adults. Additionally, over 60% of respondents stated that their workplace is easily accessible to older adults with no major barriers. Dentists in the governmental sector faced more significant barriers, while those practicing in the middle and west delta areas encountered fewer barriers than their counterparts in upper Egypt. Dentists with a higher proportion of older adult patients per day experienced the fewest barriers. Recommendations for improving knowledge and attitude include introducing mandatory courses on this topic at both the undergraduate and postgraduate levels, incorporating clinical rotations during internships and fresh graduate levels to increase contact with older adult patients, and enhancing working conditions of dental facilities. Furthermore, the introduction of mobile dental care could benefit patients who are unable to leave their homes.

### Electronic supplementary material

Below is the link to the electronic supplementary material.

Supplementary Material 1

Supplementary Material 2

## Data Availability

The datasets used and/or analyzed during the current study are available from the corresponding author on reasonable request.

## Abbreviations

WHO: World Health organization
VIF: Variance Inflation Factor
USA: United States of America
UN: United Nations
STROBE: The Strengthening the Reporting of Observational Studies in Epidemiology guidelines
ß: Regression coefficient
SD: Standard Deviation
Ph.D: Doctor of philosophy
OR: Odds Ratio
N: Number
GDP: General Dentist Practitioner
CI: Confidence Intervals

## References

[1] World health Organization. WHO: Number of people over 60 years set to double by 2050; major societal changes required. 2015 September 30, 2015 [cited 2023 March 23]; Available from: https://www.who.int/news/item/30-09-2015-who-number-of-people-over-60-years-set-to-double-by-2050-major-societal-changes-required.

[2] Economic UNDo, Social Affairs PD. *World Population Prospects 2022: Summary of Results. UN DESA/POP/2022/TR/NO. 3* 2022.

[3] Shetty P (2012). Grey matter: ageing in developing countries. The Lancet.

[4] Franceschi C (2018). The continuum of aging and age-related Diseases: common mechanisms but different rates. Front Med.

[5] Gil-Montoya JA et al. Oral health in the elderly patient and its impact on general well-being: a nonsystematic review. Clin Interv Aging. 2015;461–7. 10.2147/CIA.S54630 PMC4334280 25709420

[6] Badewy R (2021). Impact of poor oral health on community-dwelling seniors: a scoping review. Health Serv Insights.

[7] Chan AKY (2021). Common medical and dental problems of older adults: a narrative review. Geriatrics.

[8] Wyatt C, Wang D, Aleksejuniene J (2014). Incidence of dental caries among susceptible community-dwelling older adults using fluoride toothpaste: 2-year follow-up study. J Can Dent Assoc.

[9] Kassebaum N (2014). Global burden of severe periodontitis in 1990–2010: a systematic review and meta-regression. J Dent Res.

[10] Hahnel S (2014). Prevalence of xerostomia and hyposalivation and their association with quality of life in elderly patients in dependence on dental status and prosthetic rehabilitation: a pilot study. J Dent.

[11] Ahmed N et al. Comparison of canine-guided occlusion with other occlusal schemes in removable complete dentures: a systematic review. Prosthesis. 2021;3(01).

[12] Kramarow E (2007). Trends in the health of older americans, 1970–2005. Health Aff.

[13] Xavier I (2020). Geriatric dentistry curriculum in six continents. Int J Environ Res Public Health.

[14] Göstemeyer G, Baker SR, Schwendicke F (2019). Barriers and facilitators for provision of oral health care in dependent older people: a systematic review. Clin Oral Invest.

[15] Madunic D (2021). Dentists’ opinions in providing oral healthcare to elderly people: a questionnaire-based online cross-sectional survey. Int J Environ Res Public Health.

[16] Bots-VantSpijker P (2017). Dentists’ opinions on knowledge, attitudes and barriers in providing oral health care to older people living independently in the Netherlands and Flanders (Belgium). BDJ open.

[17] Tahani B, Manesh SS (2021). Knowledge, attitude and practice of dentists toward providing care to the geriatric patients. BMC Geriatr.

[18] Bots-VantSpijker PC (2016). Opinions of dentists on the barriers in providing oral health care to community‐dwelling frail older people: a questionnaire survey. Gerodontology.

[19] Anehosur GV, Nadiger RK (2012). Evaluation of understanding levels of Indian dental students’ knowledge and perceptions regarding older adults. Gerodontology.

[20] Thampan N, Sk P, James A. Awareness and knowledge on geriatric Dentistry amongst undergraduates: emphasis on the Special Care Dentistry in the Aging Realm. Indian J Public Health Res Dev. 2020;11(4).

[21] Othman AA, Yusof Z, Saub R (2014). Malaysian government dentists’ experience, willingness and barriers in providing domiciliary care for elderly people. Gerodontology.

[22] Aldhuwayhi S (2021). A comprehensive evaluation of knowledge and perceptions regarding geriatric dentistry among Saudi Arabian dental students: geriatric dentistry: Saudi students’ perspective. J Popul Ther Clin Pharmacol.

[23] Moreira AN (2012). Knowledge and attitudes of dentists regarding ageing and the elderly. Gerodontology.

[24] Statista. *Total population of Egypt as of 2022, by age group*. 2023 December 16, 2022 [cited 2023 March 23]; Available from: https://www.statista.com/statistics/1230371/total-population-of-egypt-by-age-group/.

[25] Elkady DM, Khater AGA (2023). Knowledge and attitudes toward evidence-based cariology and restorative dentistry among Egyptian dental practitioners: a cross-sectional survey. BMC Oral Health.

[26] Saleh NM (2018). Barriers affecting the Utilization of Dental Health Services among Community Dwelling older adults. Alexandria Sci Nurs J.

[27] Ettinger RL (2012). A 30-year review of a geriatric dentistry teaching programme. Gerodontology.

[28] DATAREPORTAL. Digital 2023: Egypt. 2023 [cited 2023 March 23]; Available from: https://datareportal.com/reports/digital-2023-egypt.

