# Supplementary material for: How dentists in Egypt perceive their knowledge, attitudes, and barriers they face in providing oral healthcare to geriatric patients: a cross-sectional study
Source: BMC Oral Health. 2023 Nov 29;23:947. doi: 10.1186/s12903-023-03690-9 (PMC10687915; doi:10.1186/s12903-023-03690-9)
Supplement: Supplementary file 2 — Supplementary Material 2 [file 12903_2023_3690_MOESM2_ESM.pdf]

How dentists in Egypt perceive their knowledge, attitudes and barriers they face in providing oral healthcare to geriatric patients.

Dear Fellow Dentists,

This questionnaire aims to assess knowledge, attitudes and barriers to delivery of dental care to geriatric patients.

Kindly note that

- Participation is totally voluntary and by filling the forum you are consenting to participate in the study.
- Any data collected will be maintained with the research team only and only results will be shared for publication purposes.
- You will not be asked to provide any personal details (Name, phone number or Email)

This study was approved by the ministry of health research ethics committee (Com No: 19-2022/18)

Totally agree =5 Neither =3 totally disagree =1

Section I: Demographics and professional data

1. **Gender**
   A. Male
   B. Female
2. **Academic Degree**
   A. Bachelor
   B. Master
   C. PhD
   D. Fellowship
3. **Workplace (region)**
4. **Type of practice (specialty)**
   A. Private
   B. Governmental
   C. Academic
5. **Years of experience**
   A. Less than 5 years
   B. 5 – 10 years
   C. 10 - 20 years
   D. More than 20 years
6. **Age Group**
   A. 20-29

B. 30-39
C. 40-49
D. 50-59
E. 60 or above

7. **The proportion of older patients (≥65 years) in the total number of patients per day**
   A. Less than 10 %
   B. 11 -30%
   C. More than 30%
8. **Desire to attend a course/congress on the subject of geriatric dentistry**
   A. Yes
   B. No
9. **Specialty**
   A. General dental practitioner
   B. . Prosthodontist
   C. . Orthodontist
   D. . Oral surgeon
   E. . Restorative
   F. . Pedodontics
   G. . Endodontist
   H. . Family Dentist
   I. . Preventive Dentist

Section 2: Opinion

10. **Oral health of older adults**
    A. Good
    B. Sufficient
    C. Bad
11. **Older people regularly come to dental examinations**
    A. Totally disagree
    B. Partially disagree
    C. Neither agree nor disagree
    D. Partially agree
    E. Totally agree
12. **Older people should come to the dental exam more often than younger people**
    A. Totally disagree
    B. Partially disagree
    C. Neither agree nor disagree
    D. Partially agree
    E. Totally agree

13. Providing dental care to older people is more demanding than it is to younger patients

A. Totally disagree
B. Partially disagree
C. Neither agree nor disagree
D. Partially agree
E. Totally agree

14. In the older people, poor oral health is considered a risk factor for general health problems

A. Totally disagree
B. Partially disagree
C. Neither agree nor disagree
D. Partially agree
E. Totally agree

15. Tooth loss in the older adults is an inevitable consequence of aging

A. Totally disagree
B. Partially disagree
C. Neither agree nor disagree
D. Partially agree
E. Totally agree

Section 3: Knowledge, attitudes, and barriers

16. Physical, psychological, and social aspects may influence decision-making considering oral healthcare for older people

A. Totally agree
B. Partially agree
C. Neither agree nor disagree
D. Partially disagree
E. Totally disagree

17. I have sufficient knowledge of the (adverse) effects of medicines commonly used by older people

A. Totally agree
B. Partially agree
C. Neither agree nor disagree
D. Partially disagree
E. Totally disagree

18. I am able to provide oral healthcare to cognitively impaired seniors

A. Totally agree
B. Partially agree
C. Neither agree nor disagree
D. Partially disagree
E. Totally disagree

19. **Dental medicine studies should pay more attention to the acquisition of sufficient knowledge and skills in the treatment of older people**
    A. Totally agree
    B. Partially agree
    C. Neither agree nor disagree
    D. Partially disagree
    E. Totally disagree
20. **Oral hygiene is a prerequisite for preventing oral health problems in older people**
    A. Totally agree
    B. Partially agree
    C. Neither agree nor disagree
    D. Partially disagree
    E. Totally disagree
21. **Each dentist is responsible for providing proper oral healthcare to older people who are unable to leave their home, but who have previously regularly come to their practice (with the precondition that they are their patients)**
    A. Totally agree
    B. Partially agree
    C. Neither agree nor disagree
    D. Partially disagree
    E. Totally disagree
22. **I am prepared to do a regular dental examination to an old and infirm person via a home visit**
    A. Totally agree
    B. Partially agree
    C. Neither agree nor disagree
    D. Partially disagree
    E. Totally disagree
23. **I have repeatedly experienced that at some point older, disabled people stopped coming for regular check-ups (appointments)**
    A. Totally agree
    B. Partially agree
    C. Neither agree nor disagree
    D. Partially disagree
    E. Totally disagree
24. **From the dentist's point of view, treating the vulnerable older people is not too demanding**
    A. Totally agree
    B. Partially agree
    C. Neither agree nor disagree
    D. Partially disagree

E. Totally disagree

25. Possibilities for referrals of older people with complex oral health problems to fellow specialists are limited

A. Totally agree
B. Partially agree
C. Neither agree nor disagree
D. Partially disagree
E. Totally disagree

26. Providing oral healthcare to older people is difficult because of its complexity and practical obstacles

A. Totally agree
B. Partially agree
C. Neither agree nor disagree
D. Partially disagree
E. Totally disagree

27. The reimbursement for providing oral health care to vulnerable older people is insufficient

A. Totally agree
B. Partially agree
C. Neither agree nor disagree
D. Partially disagree
E. Totally disagree

28. The institution (dental office) where I practice is easily accessible to older people (no major obstacles)

A. Totally agree
B. Partially agree
C. Neither agree nor disagree
D. Partially disagree
E. Totally disagree

29. **Usually, the provision of oral healthcare to older people involves various technical limitations**

A. Totally agree
B. Partially agree
C. Neither agree nor disagree
D. Partially disagree
E. Totally disagree

30. I find that insufficient reimbursement for the provision of oral healthcare to older people is a barrier to the professional commitment to this particular group of patients

A. Totally agree
B. Partially agree
C. Neither agree nor disagree

D. Partially disagree
E. Totally disagree

Thank you in advance
